# Clinical factors associated with arrhythmia in obstructive sleep apnea-hypopnea syndrome: a retrospective cross-sectional study

**DOI:** 10.3389/fcvm.2026.1775781

**Published:** 2026-08-05

**Authors:** Le Ma, Yibo Zhao

**Affiliations:** 1 Medical Department, Affiliated Hospital of Beihua University, Jilin, Jilin, China; 2 Department of Respiratory Medicine, Affiliated Hospital of Beihua University, Jilin, Jilin, China

**Keywords:** arrhythmia, Holter monitoring, inflammatory markers, monocyte-to-high-density lipoprotein ratio, obstructive sleep apnea-hypopnea syndrome, polysomnography

## Abstract

Obstructive sleep apnea-hypopnea syndrome (OSAHS) is frequently associated with cardiac arrhythmias; however, the clinical factors associated with concurrent arrhythmia in this population remain incompletely characterized. This study investigated factors associated with arrhythmia in patients with OSAHS, with particular focus on the monocyte-to-high-density lipoprotein cholesterol ratio (MHR). In this retrospective cross-sectional study, 451 patients with polysomnography (PSG)-confirmed OSAHS were enrolled and classified into arrhythmia and non-arrhythmia groups according to 24-hour Holter monitoring. Demographic characteristics, laboratory variables, PSG parameters, and echocardiographic indices were collected. Comparative analyses, correlation analyses, multivariable logistic regression, and receiver operating characteristic (ROC) curve analyses were performed. Arrhythmias, mainly premature atrial or ventricular contractions, were detected in 256 of 451 patients (56.8%, 256/451), and their prevalence differed significantly across OSAHS severity categories (*P* = 0.014). Compared with patients without arrhythmia, patients with arrhythmia had higher MHR and neutrophil-to-lymphocyte ratio (NLR) values and lower high-density lipoprotein cholesterol (HDL-C) levels. Multivariable analysis showed that MHR, apnea-hypopnea index (AHI), and HDL-C were independently associated with concurrent arrhythmia. Among the individual variables evaluated, MHR showed weak standalone discriminatory performance, with an area under the curve (AUC) of 0.656. MHR is a blood-derived inflammatory and lipid-related index associated with concurrent arrhythmia in patients with OSAHS. Its standalone discriminatory performance was weak; therefore, MHR should be interpreted only as an adjunctive marker for cross-sectional characterization rather than as an independent screening tool.

## 1 Introduction

Obstructive sleep apnea-hypopnea syndrome (OSAHS) is a prevalent but frequently underrecognized sleep-related breathing disorder characterized by recurrent upper airway collapse during sleep, resulting in intermittent hypoxia, sympathetic activation, and sleep fragmentation (1–5). Accumulating evidence indicates that OSAHS is associated with a broad spectrum of cardiovascular disorders, including hypertension, coronary artery disease, atrial fibrillation, and sudden cardiac death, with intermittent hypoxia, systemic inflammation, oxidative stress, and autonomic imbalance considered important contributors to cardiovascular complications (6–10). Inflammatory and cardiometabolic biomarkers have received increasing attention in studies of OSAHS-related cardiovascular abnormalities. Among these markers, the monocyte-to-high-density lipoprotein cholesterol ratio (MHR), calculated as peripheral blood monocyte count divided by serum high-density lipoprotein cholesterol (HDL-C) level, is a simple composite index reflecting the balance between monocyte-related inflammatory activity and HDL-C-related anti-inflammatory or antioxidative capacity. Because MHR is calculated from routinely measured laboratory variables rather than ordered as a standalone clinical test, its potential value lies in providing additional descriptive information when monocyte count and HDL-C are already available. However, integrated analyses examining the relationships among inflammatory markers, polysomnographic (PSG) parameters, cardiac structural features, and Holter-detected arrhythmia in patients with OSAHS remain limited. To address this gap, the present study investigated clinical, laboratory, PSG, and echocardiographic factors associated with concurrent arrhythmia in patients with OSAHS, with particular attention to MHR, and evaluated the discriminatory performance of selected variables within this cohort.

## 2 Materials and methods

### 2.1 Study population

This single-center retrospective cross-sectional study enrolled hospitalized or outpatient subjects who underwent polysomnography (PSG) at the Respiratory and Sleep Center of our hospital between January 2020 and June 2024 and were diagnosed with OSAHS. The standard diagnostic workflow at our center was as follows: patients referred for suspected OSAHS first underwent overnight PSG, and patients with PSG-confirmed OSAHS were routinely scheduled for 24-hour Holter monitoring, fasting serum biochemical testing, and transthoracic echocardiography during the same diagnostic admission or outpatient diagnostic episode. Holter monitoring was not performed solely because of arrhythmia-related symptoms; rather, it was used as part of the baseline cardiovascular assessment in patients with OSAHS. The interval between PSG and Holter monitoring was a median of 2 days (interquartile range [IQR], 1–4 days; range, 0–7 days), and patients whose Holter monitoring was performed outside the prespecified diagnostic window of 7 days after PSG were not included.

Patient screening proceeded as follows: 612 patients underwent PSG and met the diagnostic criteria for OSAHS during the study period. After excluding 76 patients without available 24-hour Holter monitoring, 38 patients without complete fasting serum biochemical data required for MHR calculation, 29 patients with prior or current OSAHS-specific treatment before completion of baseline assessments, and 18 patients meeting other exclusion criteria, 451 patients were included in the final analysis.

Patients were stratified according to the PSG-derived apnea-hypopnea index (AHI) using a uniform definition: mild OSAHS (5 ≤ AHI < 15 events/hour), moderate OSAHS (15 ≤ AHI < 30 events/hour), and severe OSAHS (AHI ≥ 30 events/hour). Based on 24-hour Holter monitoring, participants were categorized into the non-arrhythmia group or the arrhythmia group. Holter-detected rhythm abnormalities included atrial premature contractions, ventricular premature contractions, atrial fibrillation, atrial flutter, supraventricular tachycardia, ventricular tachycardia, and atrioventricular conduction block. Arrhythmias were classified according to standard ECG diagnostic criteria and current guideline-based definitions relevant to each subtype, including the 2023 ACC/AHA/ACCP/HRS atrial fibrillation guideline, the 2019 ESC supraventricular tachycardia guideline, the 2022 ESC ventricular arrhythmia guideline, and the 2021 ESC cardiac pacing guideline.

Inclusion criteria were as follows: (1) age 18–75 years; (2) PSG-confirmed OSAHS with predominantly obstructive respiratory events and AHI >= 5 events/hour; and (3) complete clinical records, serum biochemical data, PSG data, 24-hour Holter monitoring data, and echocardiographic data.

Exclusion criteria: (1) pre-existing structural heart disease, including heart failure, significant valvular heart disease, cardiomyopathy, or congenital heart disease; (2) previously documented persistent or clinically significant arrhythmias before enrollment that required ongoing treatment or could substantially affect rhythm assessment, such as persistent atrial fibrillation, high-grade atrioventricular block, or other major conduction disorders; (3) central sleep apnea or mixed sleep apnea with a predominant central component; (4) current or prior OSAHS-specific treatment before completion of baseline PSG, Holter monitoring, and blood sampling, including positive airway pressure therapy, oral appliance therapy, or upper airway surgery; (5) initiation of OSAHS treatment between PSG and Holter monitoring or between PSG and blood sampling; (6) severe hepatic or renal dysfunction, active malignancy, acute infection, autoimmune disease, or other systemic inflammatory conditions that might influence inflammatory biomarkers; and (7) incomplete clinical records or missing PSG, Holter monitoring, laboratory, or echocardiographic data.

### 2.2 Data collection and variable definitions

Demographic data included sex, age, body mass index (BMI), smoking and alcohol consumption history, systolic blood pressure (SBP), diastolic blood pressure (DBP), and histories of hypertension, diabetes mellitus, and coronary artery disease.

PSG parameters: All patients underwent standard overnight PSG for at least 7 h using the Alice PDx system (Philips Respironics, USA). The recorded parameters included AHI, defined as the total number of obstructive apneas and hypopneas per hour; lowest nocturnal oxygen saturation (lowest SpO₂); mean nocturnal oxygen saturation (mean SpO₂); and longest apnea duration. Nocturnal hypoxia was classified according to lowest SpO₂ as mild (85%–90%), moderate (80%–84%), or severe (<80%). OSAHS severity was classified as mild OSAHS (5 ≤ AHI < 15 events/hour), moderate OSAHS (15 ≤ AHI < 30 events/hour), or severe OSAHS (AHI ≥ 30 events/hour).

Biochemical and inflammatory markers: Fasting serum biochemical testing was part of the routine baseline evaluation for patients with PSG-confirmed OSAHS in our center and was not ordered only for selected symptoms. After overnight fasting from 23:00, venous blood samples were collected once at approximately 08:00 the following morning, before initiation of OSAHS-specific treatment. When blood sampling was not performed on the morning after PSG, only samples obtained within 3 days of PSG and before any OSAHS treatment were used. The analyzed parameters included white blood cell count (WBC), neutrophil count (NEUT), monocyte count (MONO), lymphocyte count (LY), interleukin-18 (IL-18), YKL-40, thymosin *β*4 (T*β*4), total cholesterol (TC), triglycerides (TG), low-density lipoprotein cholesterol (LDL-C), HDL-C, cystatin C (Cys-C), high-sensitivity C-reactive protein (hs-CRP), and routine liver and kidney function indices. MHR was not treated as a separate commercial or standard standalone clinical test; rather, it was calculated retrospectively from two routinely available laboratory variables, MONO and HDL-C, because previous cardiovascular studies have used MHR as an inflammation-lipid composite index (11–13). Because MHR may be affected by fasting status, concurrent inflammatory conditions, acute infection, and lipid metabolism, we used fasting samples obtained within 3 days of PSG and excluded patients with acute infection, autoimmune disease, malignancy, or other systemic inflammatory conditions. MHR was calculated as MONO divided by HDL-C, and the neutrophil-to-lymphocyte ratio (NLR) was calculated as NEUT divided by LY.

24-hour Holter monitoring: All included patients underwent ambulatory electrocardiographic monitoring for at least 20 h using 12-lead Holter monitors (MIC-12H-3S, Century Jinke, Beijing, China). Holter monitoring was part of the baseline cardiovascular evaluation in this retrospective cross-sectional study and was not restricted to patients reporting palpitations, syncope, chest discomfort, or other arrhythmia-related symptoms. For patients with more than one Holter recording during the diagnostic period, the recording closest to PSG and before OSAHS treatment was used. Data were manually reviewed by two senior ECG specialists. Recorded arrhythmia types included atrial premature contractions, atrial tachycardia, atrial fibrillation, ventricular premature contractions, ventricular tachycardia, and conduction block. The presence of any Holter-detected arrhythmia was used as the primary grouping criterion.

Echocardiographic parameters: Cardiac structure and function were evaluated by transthoracic echocardiography (Vivid E9, GE Healthcare). Measurements included left ventricular end-diastolic diameter (LVd), left ventricular end-systolic diameter (LVs), right ventricular diameter (RV), right atrial diameter (RA), left atrial diameter (LA), and left ventricular ejection fraction (LVEF). LVEF was assessed using the modified biplane Simpson method from apical four- and two-chamber views when image quality was adequate. Left atrial volume index (LAVI) was not included because LA volume was not consistently recorded in the retrospective echocardiography reports across the entire study period; therefore, LA diameter was retained as the available marker of LA size. All parameters were measured three times by echocardiography specialists blinded to the study groups, and the averaged values were verified by two cardiac electrophysiology experts.

OSAHS treatment status: Treatment status was reviewed from the medical records. Patients who had received continuous positive airway pressure (CPAP), bilevel positive airway pressure, oral appliance therapy, upper airway surgery, or other OSAHS-specific treatment before PSG, Holter monitoring, or blood sampling were excluded. No included patient received OSAHS-specific treatment before completion of baseline PSG, Holter monitoring, and serum biochemical assessment; therefore, the present analysis reflects untreated baseline clinical status.

### 2.3 Statistical analysis methods

All statistical analyses were performed using SPSS version 26.0 and R version 4.2.3. Continuous variables were expressed as mean +/- standard deviation (SD) for normally distributed data or median with interquartile range (IQR) for non-normally distributed data and were compared using the independent-samples t test or Mann–Whitney U test, as appropriate. Categorical variables were presented as frequencies and percentages and compared using the chi-square test or Fisher's exact test. Prior to correlation analysis, the normality of continuous variables was assessed using the Shapiro–Wilk test. Because several key variables (including AHI, HDL-C, and MHR) were not normally distributed, correlations were evaluated using Spearman's rank correlation, and Spearman's rho (*ρ*) is reported. Given the large sample size, the interpretation of correlations is based on the magnitude of the coefficient and its clinical relevance rather than solely on the P-value. Univariable analyses were performed to screen variables associated with concurrent arrhythmia in patients with OSAHS. Candidate variables included demographic characteristics, comorbidities, laboratory indices, PSG parameters, and echocardiographic measurements. Variables with *P* < 0.10 in univariable analyses were considered for entry into the multivariable logistic regression analysis. Collinearity diagnostics were performed before multivariable analysis using variance inflation factors (VIFs). Multivariable logistic regression was then used to identify variables independently associated with concurrent arrhythmia, and odds ratios (ORs) with 95% confidence intervals (CIs) were reported. Receiver operating characteristic (ROC) curve analysis was used to evaluate the discriminatory performance of selected variables, and the area under the curve (AUC) was calculated. To improve table readability, test statistics were not retained in the revised tables; group estimates and *P* values are reported. All tests were two-sided, and *P* < 0.05 was considered statistically significant. For comparisons across the three OSAHS severity groups, *post hoc* pairwise comparisons were performed when the overall test was significant. Bonferroni correction was applied to adjust for multiple pairwise comparisons, and Bonferroni-adjusted *P* values (P_adj) are reported where applicable.

## 3 Results

### 3.1 Baseline and selected clinical characteristics according to arrhythmia status

During the study period, 612 patients underwent PSG and met the diagnostic criteria for OSAHS. After excluding 76 patients without available 24-hour Holter monitoring, 38 patients without complete fasting serum biochemical data required for MHR calculation, 29 patients who had received OSAHS-specific treatment before completion of baseline assessments, and 18 patients meeting other exclusion criteria, 451 untreated patients with PSG-confirmed OSAHS were included in the final analysis. The median interval between PSG and Holter monitoring was 2 days (IQR, 1–4 days; range, 0–7 days).

**Figure 1:**
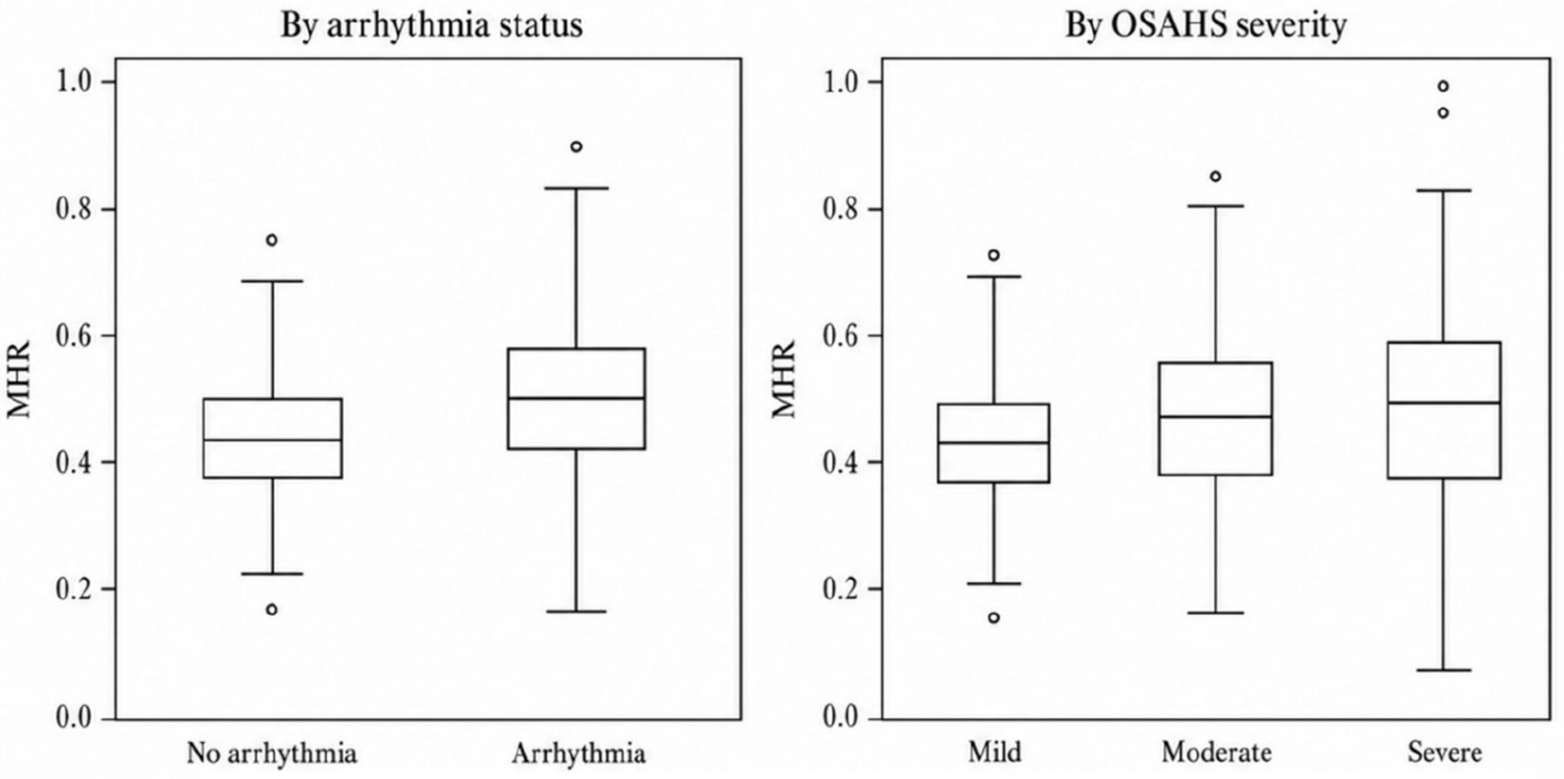
Distribution of the monocyte-to-high-density lipoprotein cholesterol ratio (MHR) in patients with OSAHS. The left panel shows MHR levels according to arrhythmia status. The right panel shows MHR levels across OSAHS severity groups.

Among the 451 included patients, 355 were male (78.7%), and the median age was 51 years (IQR, 43–58). Holter monitoring identified arrhythmias in 256 patients (56.8%), whereas 195 patients (43.2%) had no arrhythmia. Baseline demographic characteristics, including sex, age, BMI, smoking status, blood pressure, hypertension, and diabetes mellitus, were comparable between the two groups. Compared with patients without arrhythmia, patients with arrhythmia had higher AHI, lower mean SpO₂, lower HDL-C levels, and higher MHR and NLR values (Figure 1). Among echocardiographic parameters, RA diameter was slightly but significantly larger in the arrhythmia group, whereas RV diameter showed only a non-significant increasing trend (Table 1).

**Table 1:** Baseline and selected clinical characteristics according to arrhythmia status.

Parameter	OSAHS-only group (*n* = 195)	OSAHS with arrhythmia group (*n* = 256)	*P*-value
Male [n (%)]	155 (79.5%)	200 (78.1%)	0.726
Age (years)	50.74 ± 10.58	52.23 ± 8.76	0.166
BMI (kg/m^2^)	29.61 ± 5.10	29.69 ± 4.65	0.489
Smoking [n (%)]	83 (42.6%)	119 (46.5%)	0.386
SBP (mmHg)	135.18 ± 18.27	135.36 ± 16.83	0.822
DBP (mmHg)	85.89 ± 13.06	85.27 ± 13.09	0.713
Hypertension [n (%)]	142 (72.8%)	203 (79.3%)	0.111
Diabetes [n (%)]	53 (27.2%)	81 (31.6%)	0.304
AHI (events/hour)	24.34 ± 14.11	31.15 ± 18.44	<0.001*
Mean SpO₂ (%)	90.86 ± 1.41	90.59 ± 1.47	0.014*
Lowest SpO₂ (%)	76.58 ± 7.39	76.56 ± 8.03	0.574
HDL-C (mmol/L)	1.26 ± 0.21	1.15 ± 0.20	<0.001*
MHR	0.44 ± 0.10	0.50 ± 0.13	<0.001*
NLR	1.83 ± 0.56	2.01 ± 0.78	0.028*
RV (mm)	21.05 ± 2.20	21.33 ± 2.21	0.081
RA (mm)	33.98 ± 4.27	34.76 ± 3.69	0.016*

BMI, body mass index; SBP, systolic blood pressure; DBP, diastolic blood pressure; AHI, apnea-hypopnea index; HDL-C, high-density lipoprotein cholesterol; MHR, monocyte-to-HDL-C ratio; NLR, neutrophil-to-lymphocyte ratio; RV, right ventricular diameter; RA, right atrial diameter.

*P < 0.05.

### 3.2 Arrhythmia prevalence and major arrhythmia subtypes according to OSAHS severity

When patients were stratified by OSAHS severity, arrhythmias were detected in 49 of 98 patients with mild OSAHS (50.0%), 80 of 156 patients with moderate OSAHS (51.3%), and 127 of 197 patients with severe OSAHS (64.5%). The overall difference across severity groups was statistically significant (*P* = 0.014). After Bonferroni correction, arrhythmia prevalence was higher in the severe group than in the moderate group (P_adj = 0.037), whereas the severe-versus-mild comparison was borderline after correction (P_adj = 0.051).

Atrial premature contractions and ventricular premature contractions were the most common arrhythmia subtypes. Atrial premature contractions were observed in 41.8%, 32.7%, and 46.2% of patients in the mild, moderate, and severe OSAHS groups, respectively (overall *P* = 0.036). Ventricular premature contractions were observed in 22.4%, 25.0%, and 36.0% of patients, respectively (overall *P* = 0.019). However, after Bonferroni correction, only the difference in atrial premature contractions between the severe and moderate groups remained statistically significant. Atrial fibrillation and ventricular tachycardia were uncommon in this cohort (Table 2).

**Table 2:** Arrhythmia prevalence and major arrhythmia subtypes according to OSAHS severity.

Arrhythmia type [*n* (%)]	Mild group (*n* = 98)	Moderate group (*n* = 156)	Severe group (*n* = 197)	*P*-value
Any arrhythmia	49 (50.0%)	80 (51.3%)	127 (64.5%)	0.014*
Atrial premature contractions	41 (41.8%)	51 (32.7%)	91 (46.2%)	0.036*
Ventricular premature contractions	22 (22.4%)	39 (25.0%)	71 (36.0%)	0.019*
Atrial tachycardia	4 (4.1%)	19 (12.2%)	15 (7.6%)	0.067
Atrial fibrillation	1 (1.0%)	2 (1.3%)	2 (1.0%)	0.968
Ventricular tachycardia	0 (0.0%)	0 (0.0%)	2 (1.0%)	0.274
Conduction block	4 (4.1%)	8 (5.1%)	19 (9.6%)	0.117

Bonferroni-adjusted pairwise comparisons showed that overall arrhythmia prevalence was higher in the severe group than in the moderate group (P_adj = 0.037). For atrial premature contractions, the severe group differed significantly from the moderate group (P_adj = 0.031), whereas other pairwise comparisons were not significant. For ventricular premature contractions, no pairwise comparison remained statistically significant after Bonferroni correction.

*P < 0.05.

### 3.3 Selected laboratory, PSG, and echocardiographic parameters according to OSAHS severity

Selected laboratory, PSG, and echocardiographic parameters were further compared across OSAHS severity groups. HDL-C levels decreased progressively from mild to severe OSAHS, whereas MHR and NLR increased with disease severity. PSG parameters also showed expected severity-related changes: AHI and longest apnea duration increased, while mean and lowest SpO₂ decreased across the three severity groups. Because OSAHS severity was defined according to AHI, the between-group difference in AHI was expected and is reported descriptively.

Regarding echocardiographic parameters, both RV and RA diameters increased across OSAHS severity groups. After Bonferroni correction, RV and RA diameters differed significantly only between the mild and severe groups. These findings suggest that severe OSAHS was accompanied by higher inflammatory/lipid-related indices, greater hypoxic burden, and mild right-sided cardiac enlargement, although the observed structural differences were modest (Table 3).

**Table 3:** Selected laboratory, PSG, and echocardiographic parameters according to OSAHS severity.

Parameter	Mild group (*n* = 98)	Moderate group (*n* = 156)	Severe group (*n* = 197)	*P*-value
HDL-C (mmol/L)	1.27 ± 0.21	1.20 ± 0.20	1.16 ± 0.21	<0.001*
MHR	0.44 ± 0.10	0.48 ± 0.13	0.49 ± 0.16	<0.001*
NLR	1.73 ± 0.44	1.97 ± 0.71	2.02 ± 0.77	0.003*
AHI (events/hour)	9.29 ± 2.41	20.36 ± 4.25	44.03 ± 13.03	<0.001*
Mean SpO₂ (%)	91.18 ± 1.38	91.06 ± 1.46	90.12 ± 1.28	<0.001*
Lowest SpO₂ (%)	80.60 ± 6.59	78.27 ± 6.01	73.22 ± 8.13	<0.001*
Longest apnea duration (s)	26.52 ± 17.02	33.27 ± 16.92	51.11 ± 16.90	<0.001*
RV (mm)	20.56 ± 1.76	21.07 ± 2.48	21.63 ± 2.10	<0.001*
RA (mm)	33.51 ± 2.89	34.37 ± 4.13	34.93 ± 4.22	0.015*

HDL-C, high-density lipoprotein cholesterol; MHR, monocyte-to-HDL-C ratio; NLR, neutrophil-to-lymphocyte ratio; AHI, apnea-hypopnea index; RV, right ventricular diameter; RA, right atrial diameter. Bonferroni-adjusted *post hoc* comparisons showed that the mild group differed from both the moderate and severe groups for HDL-C, MHR, and NLR. AHI, lowest SpO₂, and longest apnea duration differed significantly between all pairwise severity groups. Mean SpO₂ differed significantly for severe versus mild and severe versus moderate, but not for mild versus moderate. RV and RA diameters differed significantly only between the mild and severe groups.

*P < 0.05.

### 3.4 Spearman's rank association analysis between AHI and selected clinical parameters

**Figure 2:**
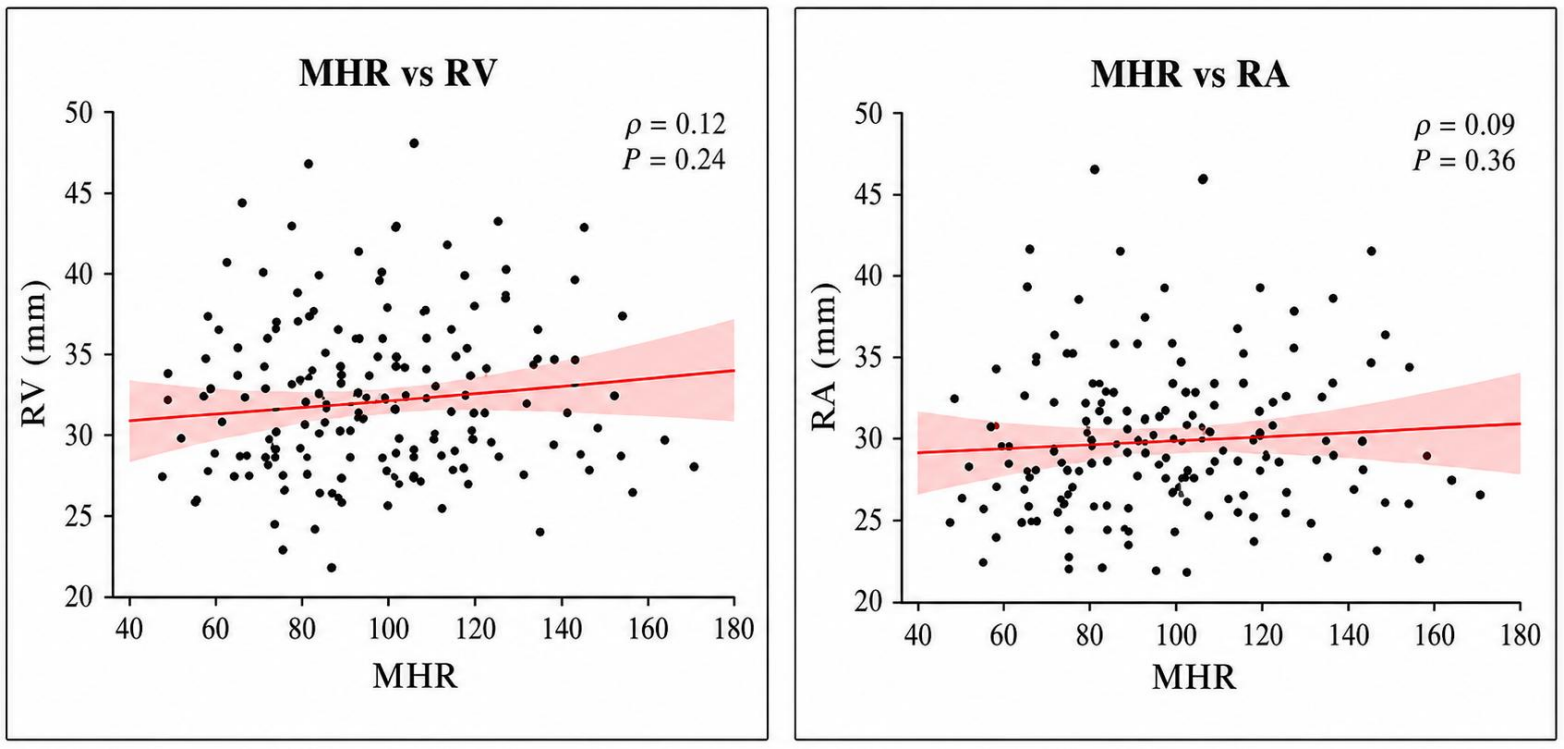
Correlation analysis between MHR and right-sided cardiac structural parameters in patients with OSAHS. Scatter plots show the relationships between MHR and right ventricular diameter (RV; left panel) and right atrial diameter (RA; right panel). The red regression line indicates the fitted linear trend, and the shaded area represents the 95% confidence interval. The correlations were weak and not statistically significant.

Spearman's rank analysis showed that the association between AHI and HDL-C was statistically significant but negligible in magnitude (*ρ* = –0.161, *P* = 0.001). Given the extremely small coefficient, this finding should not be interpreted as a clinically meaningful correlation. Similar negligible associations were observed between AHI and MHR, NLR, RV diameter, and RA diameter, with all coefficients below 0.2. Therefore, no meaningful correlation was identified between AHI and these selected clinical parameters in this cohort (Table 4). Additionally, MHR showed weak and non-significant correlations with RV diameter (*ρ* = 0.12, *P* = 0.24) and RA diameter (*ρ* = 0.09, *P* = 0.36) (Figure 2).

**Table 4:** Spearman's rank association analysis between AHI and selected clinical parameters in patients with OSAHS.

Parameter	Spearman's *ρ*	*P*-value
HDL-C (mmol/L)	−0.161	0.001*
MHR	0.159	0.001*
NLR	0.149	0.002*
RV (mm)	0.151	0.001*
RA (mm)	0.178	<0.001*

Although the *P* values reached statistical significance, all correlation coefficients were below 0.2, indicating negligible associations without clinically meaningful correlation.

*P < 0.05.

### 3.5 Multivariable logistic regression analysis

Variables with *P* < 0.10 in univariable analyses were considered for entry into the multivariable logistic regression model. After adjustment, HDL-C, MHR, and AHI remained independently associated with concurrent arrhythmia in patients with OSAHS. HDL-C showed an inverse association with arrhythmia, whereas higher MHR and higher AHI were associated with increased odds of arrhythmia. The OR for MHR was 1.049 (95% CI: 1.029–1.069), corresponding to a 4.9% higher odds of arrhythmia per 0.01-unit increment in MHR according to the scaling used in the model. NLR was significant in univariable analysis but was not retained in the final multivariable model (Table 5).

**Table 5:** Logistic regression analysis of variables associated with arrhythmia in patients with OSAHS.

Variable	Univariable *P*-value	Univariable OR	95% CI	Multivariable *P*-value	Multivariable OR	95% CI
HDL-C (mmol/L)	<0.001*	0.055	0.020–0.149	<0.001*	0.109	0.038–0.313
MHR	<0.001*	1.057	1.038–1.077	<0.001*	1.049	1.029–1.069
NLR	0.007*	1.486	1.112–1.984	—	—	—
AHI (events/hour)	<0.001*	1.026	1.014–1.038	0.002*	1.021	1.001–1.034

HDL-C, high-density lipoprotein cholesterol; MHR, monocyte-to-HDL-C ratio; NLR, neutrophil-to-lymphocyte ratio; AHI, apnea-hypopnea index; OR, odds ratio; CI, confidence interval.

*P < 0.05.

### 3.6 ROC curve analysis

**Figure 3:**
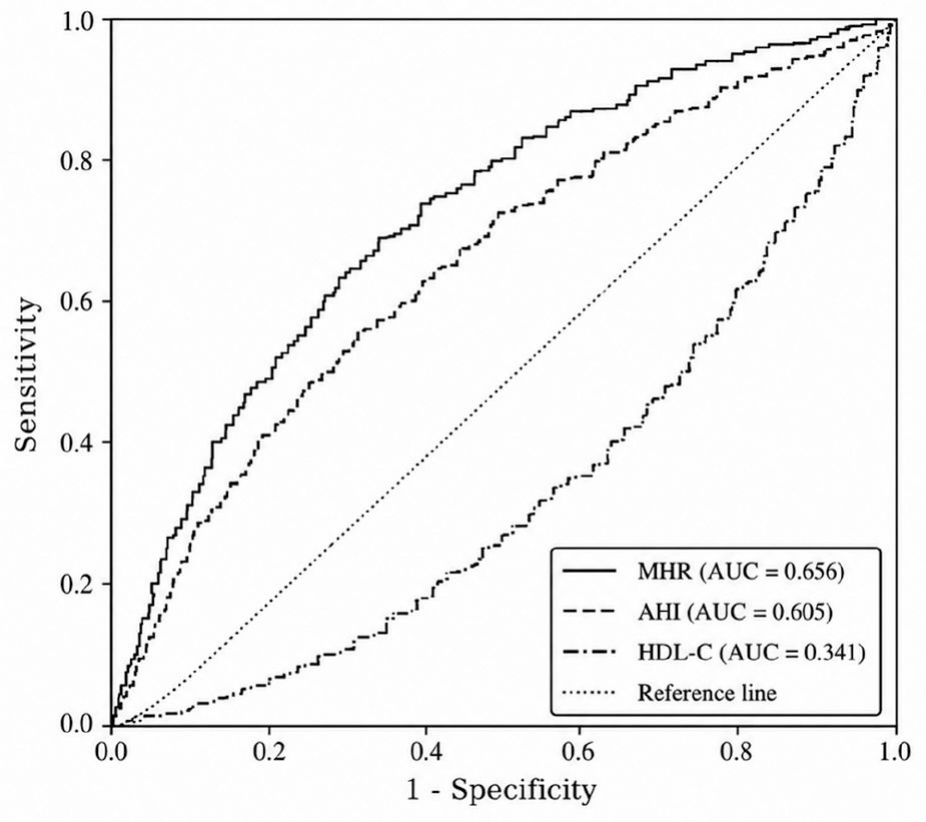
Comparative receiver operating characteristic (ROC) curves of high-density lipoprotein cholesterol (HDL-C), MHR, and AHI for discriminating arrhythmia in patients with OSAHS. MHR showed an area under the curve (AUC) of 0.656, indicating weak standalone discriminatory performance, followed by AHI (AUC = 0.605). HDL-C showed an AUC below 0.5 in its original direction, consistent with its inverse association with arrhythmia. The diagonal reference line indicates no discriminatory ability (AUC = 0.5).

ROC curve analysis was performed to evaluate the standalone discriminatory performance of HDL-C, MHR, and AHI for identifying concurrent arrhythmia in patients with OSAHS. MHR showed the highest AUC among the individual variables evaluated, with an AUC of 0.656 (95% CI: 0.605–0.707), but this still indicated weak discriminatory performance. AHI had an AUC of 0.605 (95% CI: 0.553–0.657). HDL-C yielded an AUC of 0.341 when analyzed in its original direction, which was consistent with its inverse association with arrhythmia. The optimal cutoff value for MHR was 0.427, with a sensitivity of 72.3% and a specificity of 52.3%. Overall, these results suggest that MHR may provide adjunctive information but should not be used as an independent screening tool for arrhythmia in patients with OSAHS (Table 6 and Figure 3).

**Table 6:** ROC curve analysis for discriminating arrhythmia in patients with OSAHS.

Variable	AUC	*P*-value	95% CI	Youden's index	Cut-off value	Sensitivity (%)	Specificity (%)
HDL-C (mmol/L)	0.341	<0.001*	0.291–0.392	0.008	0.705	99.2	1.5
MHR	0.656	<0.001*	0.605–0.707	0.246	0.427	72.3	52.3
AHI (events/hour)	0.605	<0.001*	0.553–0.657	0.217	40.15	31	90.8

HDL-C, high-density lipoprotein cholesterol; MHR, monocyte-to-HDL-C ratio; AHI, apnea-hypopnea index; AUC, area under the curve; CI, confidence interval. HDL-C was analyzed in its original direction, and the AUC below 0.5 reflects its inverse association with arrhythmia.

*P < 0.05.

## 4 Discussion

This study integrated PSG parameters, 24-hour Holter monitoring findings, inflammatory markers, and echocardiographic indices to evaluate factors associated with concurrent arrhythmia in patients with OSAHS. Arrhythmias were detected in 256 of 451 patients (56.8%, 256/451), and their prevalence was highest in patients with severe OSAHS. Most detected arrhythmias were PACs or PVCs, whereas sustained or clinically high-risk arrhythmias were uncommon. Traditional demographic and metabolic variables were broadly comparable between patients with and without arrhythmia. Laboratory analyses showed that patients with arrhythmia had higher MHR and NLR values and lower HDL-C levels. Echocardiographic analysis showed a modest enlargement of RA diameter in the arrhythmia group, whereas left-sided cardiac parameters and LVEF were not significantly different. In the present study, Spearman correlation coefficients between AHI and inflammatory markers as well as right-sided cardiac structural parameters were all below 0.2. Although these correlations reached statistical significance, this is largely attributable to the large sample size and they do not indicate any clinically meaningful linear relationship. Thus, OSAHS severity, as measured by AHI, shows no substantial linear association with these parameters in this cohort. In multivariable analysis, MHR, AHI, and HDL-C were independently associated with concurrent arrhythmia. ROC analysis showed that MHR had the highest AUC among the individual variables evaluated; however, its standalone discriminatory performance was weak and does not support use as an independent screening tool. The study should therefore be interpreted as an analysis of associated clinical factors rather than as a validated risk prediction model. It is important to note that the absence of meaningful linear correlations does not contradict the independent associations observed in the multivariable logistic regression model. Multivariable regression assesses the unique contribution of each variable while adjusting for others and can detect complex, non-linear, or threshold relationships that simple correlation analysis cannot capture. In this study, AHI, MHR, and HDL-C may each reflect distinct but complementary domains—hypoxic burden, inflammatory activity, and lipid-related protective capacity—that jointly contribute to the presence of arrhythmia without exhibiting strong pairwise linear correlations. Therefore, the regression findings should not be interpreted as evidence of a linear dose–response relationship, and the negligible correlations further reinforce the notion that these variables carry independent, albeit modest, information.

The findings of this study are broadly consistent with previous evidence that rhythm abnormalities are observed in patients with OSAHS (14–19). However, AF and ventricular tachycardia were uncommon in the present cohort; therefore, our results should be interpreted as describing mainly premature atrial and ventricular contractions rather than confirming AF- or ventricular tachycardia-specific associations. Regarding biomarkers, previous studies have reported the prognostic value of MHR in coronary artery disease and other cardiovascular populations (11–13). Our findings suggest that MHR may also be relevant as an associated biomarker in patients with OSAHS and concurrent Holter-detected arrhythmia. In addition, the observed enlargement of RA diameter is compatible with prior reports suggesting that OSAHS-related right-sided cardiac remodeling may be associated with arrhythmogenic susceptibility (20–22).

The low prevalence of atrial fibrillation (AF) in this cohort also warrants cautious interpretation. AF was detected in only 5 of 451 patients (1.1%, 5/451), which may be lower than expected in a predominantly male, overweight or obese OSAHS population. Several factors may explain this finding. First, arrhythmia status was determined using a single 24-hour Holter recording, which may underestimate intermittent paroxysmal AF. Second, patients with previously documented persistent or clinically significant arrhythmias requiring ongoing treatment, including persistent AF, were excluded to reduce confounding when evaluating concurrent rhythm abnormalities in the baseline OSAHS assessment. This exclusion likely removed patients with higher AF burden. Third, the composite arrhythmia endpoint in this study was driven primarily by PACs and PVCs rather than AF; therefore, the present findings should not be generalized to AF-specific prediction.

Several mechanisms may underlie the association between OSAHS and arrhythmia reported in previous studies. Intermittent hypoxia, a core feature of OSAHS, has been linked to oxidative stress and activation of inflammatory signaling pathways, including nuclear factor kappa-B (NF-kB), as well as monocyte recruitment and pro-inflammatory cytokine release (23–25, 33, 34, 36). An elevated MHR may reflect both increased monocyte-mediated inflammatory activity and reduced HDL-C-related anti-inflammatory capacity. Recurrent hypoxia and hypercapnia have also been associated with autonomic imbalance, reduced HRV, and myocardial electrical instability (26–28). With respect to right-sided cardiac enlargement, the present findings should be interpreted mainly in relation to OSAHS-related pulmonary vascular load, intrathoracic pressure swings, and mechanical stretch rather than as evidence that inflammation directly caused RA dilation. Prior studies have linked OSAHS with right-heart structural remodeling and pulmonary hypertension-related right ventricular remodeling (21, 29), and pressure overload or stretch-related right-sided remodeling may contribute to electrical instability (30–32). Although inflammation may coexist with these processes, this study did not show a significant linear correlation between MHR and RA or RV diameter; therefore, we do not infer that inflammation directly caused RA enlargement. These mechanisms are discussed as biologically plausible explanations from prior literature and should not be interpreted as causal conclusions from the present retrospective cross-sectional analysis. Furthermore, the negligible Spearman correlations observed between AHI and the inflammatory/structural parameters indicate that, at least in this cross-sectional snapshot, the severity of intermittent hypoxia does not translate into a proportional increase in these circulating or echocardiographic measures. This underscores the complexity of OSAHS-related pathophysiology and the likelihood that other unmeasured factors (e.g., individual hypoxic burden, arousal frequency, genetic susceptibility) modulate these relationships.

From a clinical perspective, the findings should not be interpreted as indicating that most Holter-detected arrhythmias in this cohort require direct antiarrhythmic treatment. Because the arrhythmia outcome was driven mainly by premature atrial and ventricular contractions, these findings may reflect altered autonomic tone, hypoxic burden, hemodynamic stress, or early structural remodeling in some patients with OSAHS. Rather, MHR may serve as an adjunctive marker when interpreted together with clinical symptoms, PSG parameters, cardiovascular comorbidities, and echocardiographic findings. Potential clinical actions include optimization of OSAHS management, assessment of cardiovascular comorbidities, and selective ECG or Holter follow-up in patients with relevant symptoms or cardiovascular risk factors. Future prospective studies are needed to determine whether MHR-guided assessment provides incremental clinical benefit beyond standard clinical evaluation.

The present findings may have several implications for real-life clinical practice. First, patients with OSAHS, especially those with severe disease, should not be evaluated only from the perspective of sleep-disordered breathing. Because arrhythmias in this cohort were mainly represented by premature atrial and ventricular contractions, our results support a more integrated clinical assessment that combines PSG findings, symptoms, cardiovascular comorbidities, routinely available laboratory markers, and basic cardiac evaluation. In daily practice, elevated MHR should not be used as an independent screening tool or treatment trigger, given its weak standalone discriminatory performance. However, when monocyte count and HDL-C are already available, MHR may provide additional contextual information to help clinicians identify patients who may benefit from more careful cardiovascular review, optimization of OSAHS management, and selective ECG or Holter follow-up when clinically indicated. This is consistent with the broader view proposed by Sircu et al. (10), who emphasized that OSAS is a systemic disorder associated with cardiovascular, metabolic, cerebrovascular, renal, and neuropsychiatric comorbidities, and that timely screening and management of comorbid conditions are important components of OSAS care.

CPAP remains the cornerstone therapy for symptomatic or clinically significant OSAS and should be considered an upstream intervention targeting intermittent hypoxia, sympathetic activation, sleep fragmentation, oxidative stress, and inflammation (35). From our perspective, CPAP may help reduce the arrhythmogenic substrate in selected patients with OSAS by improving nocturnal oxygenation and reducing autonomic instability and cardiometabolic stress. Nevertheless, the present retrospective cross-sectional study did not evaluate CPAP treatment or longitudinal arrhythmia outcomes; therefore, our data cannot prove that CPAP reduces arrhythmia incidence. The available literature also suggests that the effect of CPAP on arrhythmias is heterogeneous: some observational studies support a beneficial role, especially in reducing atrial fibrillation recurrence after rhythm-control therapy, whereas randomized evidence remains limited and not uniformly positive. Therefore, CPAP should be interpreted as an important component of comprehensive risk-factor management rather than as a substitute for rhythm-specific therapies.

In clinical decision-making, CPAP, catheter ablation, and implantable cardioverter-defibrillators should be viewed as complementary rather than competing strategies. CPAP targets the upstream OSAS-related pathophysiological drivers, whereas catheter ablation is used to treat selected symptomatic or recurrent atrial arrhythmias, particularly atrial fibrillation, and implantable cardioverter-defibrillators are reserved for patients with guideline-based indications for prevention of sudden cardiac death or treatment of life-threatening ventricular arrhythmias. For OSAS patients with atrial fibrillation, untreated sleep apnea may reduce the effectiveness of rhythm-control strategies, and CPAP may be helpful as part of integrated risk-factor modification. However, CPAP should not delay or replace catheter ablation when ablation is clinically indicated. Similarly, in patients with malignant ventricular arrhythmias or high sudden-death risk, CPAP cannot replace an implantable cardioverter-defibrillator. Instead, CPAP should be used to reduce modifiable OSAS-related stressors while standard electrophysiological therapies are applied according to established clinical indications. Because most arrhythmias in the present cohort were premature beats and high-risk arrhythmias were uncommon, prospective studies are needed to determine whether CPAP-guided management can reduce arrhythmia burden or improve long-term cardiovascular outcomes in patients with OSAHS.

This study has several limitations. First, this was a single-center retrospective cross-sectional study, which is inherently subject to selection bias and does not permit inference regarding temporal sequence or causality between OSAHS-related variables and concurrent arrhythmia. Although Holter monitoring and fasting biochemical testing were part of the routine diagnostic workflow in our center, only patients with available PSG, Holter monitoring, and serum biochemical data could be included. Therefore, selection bias related to missing Holter or laboratory data cannot be fully excluded, and the numbers excluded for these reasons have been reported in the Study Population and Results sections. Second, the study outcome was based on Holter-detected arrhythmias recorded during the overall analyzable monitoring period, rather than a dedicated sleep-period analysis. Because nocturnal hypoxemia and autonomic instability are most relevant to OSAHS pathophysiology, sleep-specific rhythm assessment may provide more precise information in future studies. Third, the composite outcome of “any arrhythmia” included rhythm abnormalities with different pathophysiological mechanisms and clinical implications, and the overall prevalence was driven largely by premature atrial and ventricular beats rather than by sustained or clinically high-risk arrhythmias. This heterogeneity should be considered when interpreting the findings. Fourth, although several inflammatory and structural variables were evaluated, some potentially relevant mechanistic domains, such as autonomic function, HRV, circadian rhythm changes, and detailed electrophysiological parameters, were not systematically assessed. In addition, several exploratory biomarkers were not fully incorporated into the final analytical framework. Fifth, LAVI could not be reported because LA volume was not consistently available in retrospective echocardiography reports across the study period; therefore, LA diameter was used as the available LA size parameter. Sixth, MHR is a calculated index and may vary with fasting status, acute or chronic inflammatory conditions, infection, and lipid metabolism. Although fasting sampling and exclusion of patients with acute infection, malignancy, autoimmune disease, or other systemic inflammatory conditions reduced some sources of variability, residual biological and measurement variability cannot be excluded. Seventh, the multivariable logistic regression analysis was developed and internally evaluated within a single dataset, and the cutoff values derived using the Youden index are sample-specific. Accordingly, the observed associations, discriminatory performance, and proposed thresholds require external validation in independent cohorts before broader clinical application. In addition, AF prevalence may have been underestimated because a single 24-hour Holter recording was used and because patients with pre-existing persistent or clinically significant arrhythmias, including persistent AF, were excluded by design. Finally, because most detected rhythm abnormalities were premature beats and the AUC of MHR was weak, the clinical implications of using MHR to guide additional rhythm monitoring remain uncertain.

## 5 Conclusions

This study systematically evaluated arrhythmia profiles and associated clinical factors in patients with OSAHS. Arrhythmias were common in this cohort and were most frequently represented by premature atrial and ventricular contractions. Arrhythmia prevalence was highest among patients with severe OSAHS. MHR, AHI, and HDL-C were independently associated with concurrent arrhythmia, but MHR showed only weak standalone discriminatory performance. These findings suggest that MHR, a calculated index derived from monocyte count and HDL-C rather than a standalone clinical test, may serve as an adjunctive marker for cross-sectional characterization of patients with OSAHS, pending validation in independent prospective cohorts.

## Data Availability

The original contributions presented in the study are included in the article/Supplementary Material, further inquiries can be directed to the corresponding author.
